# Doping im Spannungsfeld zwischen Betrug, Gesundheitsschutz und Verantwortung

**DOI:** 10.1007/s00103-026-04260-4

**Published:** 2026-07-01

**Authors:** Uwe Koch-Gromus

**Affiliations:** Institut und Poliklinik für Medizinische Psychologie, Universitätsklinikum Hamburg-Eppendorf, Martinistr. 52, 20246 Hamburg, Deutschland

Viele Menschen empfinden Sport als bereichernd, sei es durch aktive Teilnahme oder als Zuschauer. Wenn Sportereignisse kritisch diskutiert werden, stehen seit längerer Zeit vor allem 2 Themen im Mittelpunkt, nämlich die Kommerzialisierung bestimmter Bereiche des Wettkampfsportes und Doping. Letzteres ist Gegenstand dieses Themenheftes.

Es gibt inzwischen zahlreiche meist nur gering voneinander abweichende Definitionen von Doping. So definierte der Europarat 1963 Doping als „die Verabreichung oder den Gebrauch körperfremder Substanzen in jeder Form und physiologischer Substanzen in abnormaler Form oder auf abnormalem Wege an gesunde Personen mit dem einzigen Ziel der künstlichen und unfairen Steigerung der Leistung für den Wettkampf“. Damit rückt Doping in unmittelbare Nähe des Sportbetruges. Die Arten des Dopings sind vielfältig und haben sich über die Zeit stetig erweitert. Zu nennen sind u. a. Stimulanzien und Narkotika, anabole Steroide, Peptid- und Glykoproteinhormone und metabolische Modulatoren. Zunehmend relevant sind auch die Methoden des Gendopings. Doping wird in der Öffentlichkeit eng mit dem Spitzensport in Verbindung gebracht. Diskutiert werden in diesem Kontext vor allem mögliche körperliche und psychische Folgen. Diese reichen von Abhängigkeiten über chronische Organschäden bis hin zum Tod. Traurige Berühmtheit erlangte der Engländer Tom Simpson 1967, der auf einer Bergetappe der Tour de France infolge extremer Dehydration und Erschöpfung verstarb. Er stand dabei unter Einwirkung von Amphetamin und Alkohol.

Inzwischen gibt es eine lange Liste von Athletinnen und Athleten, die nachweislich gedopt haben; nicht selten handelt es sich dabei um Personen mit wiederholten Dopingverstößen. Zu nennen sind etwa die besonders erfolgreichen Radrennfahrer Lance Armstrong und Jan Ullrich, die zunächst Doping bestritten, später jedoch systematisches Doping einräumten. Nahezu jede Sportart ist betroffen. In vielen Fällen zog sich die Beweisführung über Jahre hin, oft ohne abschließende Klärung. Erwähnt sei hier der Langstreckenläufer Dieter Baumann oder erst jüngst der Fall des Tennisspielers Jannick Sinner.

Dopingskandale stellen ein stark medienwirksames Thema dar. Die politische Dimension des Dopings wurde besonders deutlich, als die DDR und andere osteuropäische Staaten mit guten Belegen des sog. Staatsdopings beschuldigt wurden. Auch in westlichen Nationen hatte Doping eine nicht unwesentliche Bedeutung. Es wurde nicht flächendeckend, sondern eher in „privaten Zirkeln“ praktiziert, oftmals allerdings mit Duldung durch politische Institutionen.

Inzwischen wurden zahlreiche Methoden zum Nachweis von Doping entwickelt und rechtliche Voraussetzungen zur Kontrolle von Doping geschaffen. Nationale und internationale Einrichtungen, insbes. die Welt-Anti-Doping-Agentur (WADA) und die Nationale Anti-Doping Agentur Deutschland (NADA), nehmen Aufgaben der Dopingkontrolle wahr. Dabei geraten diese Einrichtungen selbst immer wieder in die Kritik und ein kontinuierlicher und konstruktiver Dialog der beteiligten Parteien scheint hier von essenzieller Bedeutung zu sein. Die Ende Mai 2026 in Las Vegas (USA) ausgetragenen Enhanced Games haben die mediale und gesellschaftliche Debatte über Doping und Leistungssteigerung im Sport erneut aufgegriffen und weiter befeuert. Im Fokus stand dabei insbesondere, dass Athletinnen und Athleten im Kampf um hohe Preisgelder leistungssteigernde Mittel einsetzen konnten, ohne klassische Dopingkontrollen befürchten zu müssen. Die Veranstaltung hat damit bestehende Diskussionen über die Grenzen sportlicher Leistungssteigerung und die Vorbildfunktion des Spitzensports für junge Menschen weiter zugespitzt.

Doping ist allerdings keineswegs nur ein Problem des Spitzensports. Es gibt eindeutige Belege, dass es auch im Breitensport häufig – und hier besonders unkontrolliert – zur Anwendung kommt. Auch außerhalb des Sports ist in der Bevölkerung die Einnahme von Stimulanzien und anderen Substanzen zur Leistungssteigerung oder zur Verbesserung des subjektiven Wohlbefindens verbreitet, etwa im Arbeitsleben, in Prüfungssituationen, in der Freizeit oder im Sexualleben. In gewisser Hinsicht kann dies mit Doping verglichen werden.

Die Aufklärung vieler Dopingfälle zeigt sehr deutlich, dass Ärztinnen und Ärzte sowie medizinische Institutionen häufig am Dopinggeschehen mitgewirkt haben. Nicht selten waren sie Teil des Systems. Oder pointierter formuliert: Ohne medizinischen Sachverstand gibt es kein systematisches Doping. Dieser ist erforderlich, um Doping geheim zu praktizieren und Spuren möglichst sicher zu verbergen. Die Mitwirkung beim systematischen Doping ist mit dem ärztlichen Ethos unvereinbar. Eher selten führten strafrechtliche Ermittlungen gegen Ärztinnen und Ärzte wegen Unterstützung des Dopings mit möglichem Schaden für Athletinnen und Athleten zum Erfolg.

Vor diesem Hintergrund behandelt das Themenheft mit insgesamt 8 Beiträgen ausgewählte Fragestellungen zu Doping im Spitzensport und in weiteren Lebensbereichen. Thematisch lassen sich die ausgewählten Beiträge in 2 Inhaltsbereiche untergliedern. In den ersten 4 Artikeln werden gesellschaftliche Aspekte des Dopings behandelt. Im zweiten Themenkomplex geht es um Erscheinungsformen und -felder des Dopings.

Im Eingangsbeitrag zeichnet *S. Wassong* die institutionellen Entwicklungslinien der Antidopingpolitik des Internationalen Olympischen Komitees (IOC) aus historischer und aktueller Perspektive nach. Den Anti-Doping-Institutionen, die unter dem Einfluss des IOC (WADA, NADA, International Testing Agency – ITA) entstanden, wird dabei besondere Bedeutung zugeschrieben.

Der sich anschließende Artikel von *H. Striegel* zeigt die sportrechtlichen Rahmenbedingungen im Umgang mit Dopingsubstanzen, einschließlich der damit verbundenen Strafen und Sanktionen, auf. Für Deutschland relevant ist dabei das 2015 erlassene Anti-Doping-Gesetz. Darüber hinaus diskutiert der Beitrag die Auswirkungen von Doping auf die Gesellschaft und Sportkultur. Im Mittelpunkt stehen dabei Fairness, Chancengleichheit und Integrität des sportlichen Wettbewerbs, gesundheitliche Risiken sowie Strategien der Dopingprävention.

Der medialen Berichterstattung kommt bei der Entwicklung gesellschaftlicher Einstellungen zum Doping besondere Bedeutung zu. Die Aufdeckung von Dopingfällen durch die Medien hat sicher dazu beigetragen, die negativen Folgewirkungen von Doping in Hinblick auf gesundheitliche Folgen oder Unfairness im Wettbewerb in die Öffentlichkeit zu bringen, und dürfte auch die Schaffung von Kontrollinstitutionen forciert haben. Andererseits hat der gelegentlich durch Sensationslust und Falschmeldungen geprägte journalistische Umgang mit einzelnen Dopingfällen genau diesem Anliegen geschadet. Wie Spitzensportlerinnen und -sportler Berichte über Doping auf ihr eigenes Verhalten einschätzen, beschreibt der Text von *M. Schaffrath und T. Schulz.*

Der sich anschließende Beitrag von *C. Spitzer und B. Bley* setzt sich mit den gesundheitlichen Folgen, der Aufarbeitung und dem rechtlichen Ausgleich des Staatsdopings im DDR-Leistungssport auseinander. Die Autoren beschreiben Mechanismen des staatlich gesteuerten Zwangsdopings, die Vielfalt und Schwere der gesundheitlichen, psychischen und sozialen Folgen und die Grenzen der Aufarbeitung des Dopings.

Der zweite Themenkomplex des Heftes befasst sich in 4 weiteren Beiträgen mit Doping in verschieden Handlungsfeldern: im Leistungssport, im Breitensport, in einer „sanften“ Sportart (Schach) und im Alltag bzw. in der Arbeitswelt. *M. Thevis et al.* stellen das breite Spektrum aktueller analytischer Verfahren dar, die unter anderem zum Nachweis gentherapeutischer Substanzen und anderer unerlaubter Methoden der Leistungssteigerung im Leistungssport eingesetzt werden. Die Autorinnen und Autoren sehen vor dem Hintergrund der hohen Dynamik der Entwicklung die Notwendigkeit, neue Nachweisverfahren in der Doping-Routine-Analytik zu entwickeln. Aus Sicht des Verfassers des Editorials reicht es allerdings nicht aus, neuere und bessere Methoden zum Aufspüren des Dopings zu entwickeln. Es muss auch sichergestellt werden, dass diese wirksam implementiert und regelmäßig kontrolliert werden und die Finanzierung der Maßnahmen künftig gesichert wird.

Dass Doping keineswegs nur ein Problem des Leistungssportes ist, zeigt der Beitrag von *P. Dietz et al., *der sich mit der Rolle leistungssteigernder Substanzen im Breitensport auseinandersetzt. Die Forschung in diesem Bereich ist mit besonderen methodischen Problemen konfrontiert und hat in den letzten Jahren eigene Strategien entwickelt. Die Autoren kommen zu der Schlussfolgerung, dass der Konsum leistungssteigernder Substanzen im Breitensport kein Randphänomen ist.

In der Regel wird Doping mit intensiven körperlichen Aktivitäten in Verbindung gebracht. Der folgende Beitrag zeigt jedoch, dass vergleichbare Phänomene auch in Sportarten auftreten können, die weniger körperbetont sind. So beschreibt *T. E. Wessendorf, *dass im Schachsport der Gebrauch leistungssteigernder Substanzen vorkommt, hier vor allem Mittel zur kognitiven Leistungssteigerung, und es Hinweise gibt, dass die Einnahme von Stimulanzien zu einem messbar besseren Ergebnis führen kann.

Im Artikel von *S. Milin et al.* werden in einer^.^ narrativen Übersichtsarbeit Kontexte, Motive, Substanzgruppen, Nutzenannahmen und Risiken analysiert. Die Studien zeigen, dass der Konsum primär der kurzfristigen Sicherung einzelner Funktionsbereiche dient. Zentrale Kontexte sind Müdigkeit und hohe kognitive Anforderungen, affektive Belastung und Stress sowie körperliche Beschwerden.

Liebe Leserinnen und liebe Leser, ich hoffe, dass es mit diesem Themenheft gelungen ist, ein breitgefächertes Bild des aktuellen Diskussionstands zum Thema Doping zu vermitteln. Das Themenheft zeigt, dass Doping weit über den Spitzensport hinausweist und grundlegende Fragen nach Leistung, Fairness und gesellschaftlichem Erfolgsdruck berührt. Ich danke Eberhard Hildt (Paul Ehrlich Institut) für die Anregung zu diesem Themenheft und seine in der Initialphase beigesteuerten Ideen. Sehr herzlich bedanken möchte ich mich auch bei Mario Thevis, Klaus-Michael Braumann, Klaus Püschel, Holger Schulz und Martin Härter für ihre Anregungen bei der Gestaltung des Editorials. Allen Autorinnen und Autoren danke ich für die gute Zusammenarbeit. Ich wünsche Ihnen, liebe Leserinnen und Leser, eine anregende Lektüre.

## Data Availability

Alle dieser Arbeit zugrunde liegenden Daten sind in diesem Artikel enthalten.

